# Rapid full-wave phase aberration correction method for transcranial high-intensity focused ultrasound therapies

**DOI:** 10.1186/s40349-016-0074-7

**Published:** 2016-12-08

**Authors:** Scott Almquist, Dennis L. Parker, Douglas A. Christensen

**Affiliations:** 1 School of Computing, University of Utah, Salt Lake City, UT USA; 2 Utah Center for Advanced Imaging Research, Department of Radiology, University of Utah, Salt Lake City, UT USA; 3 Department of Radiology, University of Utah, Salt Lake City, UT USA; 4 Department of Bioengineering, University of Utah, Salt Lake City, UT USA; 5 Department of Electrical and Computer Engineering, University of Utah, Salt Lake City, UT USA

**Keywords:** MRgHIFU, Transcranial HIFU, Phase aberrations, Ultrasound simulation methods

## Abstract

**Background:**

Non-invasive high-intensity focused ultrasound (HIFU) can be used to treat a variety of disorders, including those in the brain. However, the differences in acoustic properties between the skull and the surrounding soft tissue cause aberrations in the path of the ultrasonic beam, hindering or preventing treatment.

**Methods:**

We present a method for correcting these aberrations that is fast, full-wave, and allows for corrections at multiple treatment locations. The method is simulation-based: an acoustic model is built based on high-resolution CT scans, and simulations are performed using the hybrid angular spectrum (HAS) method to determine the phases needed for correction.

**Results:**

Computation of corrections for clinically applicable resolutions can be achieved in approximately 15 min. Experimental results with a plastic model designed to mimic the aberrations caused by the skull show that the method can recover 95 % of the peak pressure obtained using hydrophone-based time-reversal methods. Testing using an ex vivo human skull flap resulted in recovering up to 70 % of the peak pressure at the focus and 61 % when steering (representing, respectively, a 1.52- and 1.19-fold increase in the peak pressure over the uncorrected case). Additionally, combining the phase correction method with rapid HAS simulations allows evaluation of such treatment metrics as the effect of misregistration on resulting pressure levels.

**Conclusions:**

The method presented here is able to rapidly compute phases required to improve ultrasound focusing through the skull at multiple treatment locations. Combining phase correction with rapid simulation techniques allows for evaluation of various treatment metrics such as the effect of steering on pressure levels. Since the method computes 3D pressure patterns, it may also be suitable for predicting off-focus hot spots during treatments—a primary concern for transcranial HIFU. Additionally, the plastic-skull method presented here may be a useful tool in evaluating the effectiveness of phase correction methods.

## Background

High-intensity focused ultrasound (HIFU) is becoming prominent as a treatment method for a large number of diseases. The lack of ionizing radiation and non-invasive nature of HIFU makes it well suited as a primary or supplementary treatment for uterine fibroids [1], prostate cancer [2, 3], bone metastasis [4], and other afflictions [5]. In the brain, transcranial HIFU is being investigated for neurostimulation [6], to treat movement disorders [7] and gliomas [8], and may be useful in other treatments [9].

Transcranial HIFU, however, faces challenges not present for treatments in other areas in the body. The large attenuation (10 dB on average at 0.5 MHz and 20 dB at 1.5 MHz [10]) caused by the skull requires the use of large-aperture transducers to evenly spread the power over the skull to prevent skin burns [11]. Additionally, the large difference in acoustic properties of the skull and surrounding soft tissues—notably the speed of sound, which is approximately 2740 m/s in the skull [10] and near 1500 m/s in most soft tissues [12]—introduces phase aberrations that cause the focus to be distorted, displaced, and of lower intensity. Nevertheless, aberrations can be corrected by phasing the elements of a phased-array transducer such that constructive interference is achieved at the intended treatment location.

There exist several methods for determining the phases required to correct for aberrations [13]. The methods can be broadly classified into time-reversal, energy-based, and simulation-based methods. One version of the time-reversal method [14] relies on an implantable hydrophone to measure the phases from each element in order to determine the inverse phase for correction. Although this offers excellent correction, the invasive nature makes it inappropriate for HIFU treatments. Energy-based methods use imaging to determine the effect of the skull on the ultrasonic beam. For example, cavitation can create shock waves that can be imaged noninvasively (“ultrasonic stars” [15]), but the energy required to create cavitation and the bubbles themselves may pose a risk to the patient. Methods based on MR acoustic radiation force imaging [16] avoid cavitation but may require more time to converge to the correct phases. Simulation-based methods predict the phases through numerical simulation. Often, these simulations are based on acoustic properties derived from a CT scan [17], although using MR images of the skull obtained using UTE sequences may be possible [18]. Finite-difference time-domain (FDTD) simulations took about 2 h to compute phase corrections [19]. Faster simulations can be achieved by simplifying the acoustic models used [20], but the resulting phases will have reduced accuracy. It has been shown that full-wave simulation methods have an improved accuracy over ray tracing and can be computed within minutes [21]; however, the technique used there was limited to a single point of correction and did not include experimental results. Another work [22] has also shown full-wave methods to produce superior results compared to ray tracing at the expense of computational time (hours vs. seconds). A review of phase correction methods can be found in [13].

In this paper, we introduce a simulation-based full-wave phase aberration correction method. The method leverages the speed of the hybrid angular spectrum (HAS) method of acoustic simulation [23] in order to rapidly determine the phases required. HAS is implemented on a graphics processing unit (GPU) in order to quickly determine the acoustic pressure field in a 3D volume for each element, allowing for phase correction at multiple treatment locations with negligible increase in computational time.

We demonstrate the method’s effectiveness experimentally using two models. First, we correct the phase aberrations for a 3D-printed plastic aberrator model that is designed to simulate the phase aberrations caused by a human skull, but for which the acoustic properties can be accurately determined. Second, phase corrections are performed using a section of an ex vivo human skull. We further expand on the capabilities of this method by combining it with rapid HAS simulations to demonstrate and evaluate the effects of transducer/model misregistration and the effects of steering on pressure levels with reasonable calculation times.

## Methods

### Simulation technique

The phase aberration correction method in this paper simulates acoustic pressure patterns using the HAS method [23]. HAS alternates between the space and spatial-frequency domains as it propagates a steady state wave through transverse sections of a model to simulate 3D ultrasonic beam patterns; it simulates refraction, reflection, and absorption. HAS demonstrates a computational speed increase over other simulation methods. For example, a 301 × 301 × 300-voxel model with 0.15-mm isotropic resolution with three tissue types computed on a 2.67-GHz i7-Core Windows desktop with 12 GB of RAM using MATLAB 7.10 took 46 s in HAS versus 467 min using a FDTD technique using the same input source [23], though this did not exploit parallelism for either HAS or the FDTD technique. The HAS method models radiating boundary conditions, which was also modeled in the FDTD simulation.

The mathematical basis for the HAS approach is a discrete solution for the steady state Helmholtz equation

1 $$ {\nabla}^2A+{k}^2A=0, $$

where *A* is a pressure field and *k* is the wave number. Using the pressure *p*_*n*-1_(*x*, *y*), which is the pressure pattern at the entrance to a given slice of the model at layer *n*, the space-domain step for HAS is calculated as follows:

2 $$ {p}_n^{\mathit{\hbox{'}}}\left(x,y\right)={p}_{n-1}\left(x,y\right){e}^{j\varDelta b\left(x,y\right)r\mathit{\hbox{'}}}{e}^{-{a}_n\left(x,y\right)r}. $$

Here, *r* is the oblique distance between the slices taken at angles of the plane wave components and *r*′ is the perpendicular distance between the angular component and the *z*-axis. *Δb*(*x*, *y*) is a propagation term that represents the difference between a particular voxel’s propagation phase and the average propagation phase used in the spatial-frequency domain step (next). *a*_*n*_(*x*, *y*) is the attenuation for each voxel. After the space-domain step, the Fourier transform of *p*_*n*_ *′*(*x*, *y*) is computed yielding *A′*(*α/λ*, *β/λ*). In the spatial-frequency domain, the pattern is propagated using a transfer function

3 $$ {A}_n\left(\frac{\alpha }{\lambda },\frac{\beta }{\lambda}\right)={p}_n^{\mathit{\hbox{'}}}\left(\frac{\alpha }{\lambda },\frac{\beta }{\lambda}\right){e}^{j{b}_n{\textstyle \hbox{'}}\sqrt{1-{\alpha}^2-{\beta}^2}\Delta z}, $$

where *α* and *β* are the directional cosines (*α = λf*_*x*_ and *β = λf*_*y*_), *b*_*n*_ *′* is an average propagation constant across the plane, and *Δz* is the propagation distance from one plane to the next. The inverse Fourier transform of *A*_*n*_ will be the pressure pattern at the face of the next plane, *p*_*n*_. The process can be repeated for each slice along the model.

HAS is able to account for inhomogeneities at a voxel-by-voxel level and, if needed, is able to account for reflections by calculating the impedance mismatch between voxels during the space-domain step. Although HAS is capable of modeling scattering [24], it was not employed for these simulations. HAS does not currently model nonlinearities or shear waves.

**Fig. 1:**
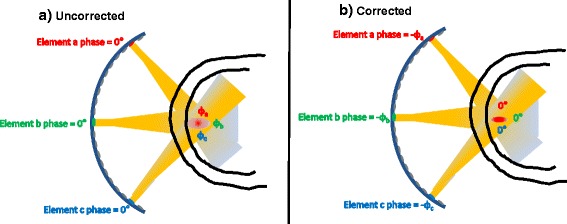
Phase correction method. **a** Ultrasonic waves are simulated from each individual element assuming zero phase, producing a 3D array of phases corresponding to any desired treatment location. Three arbitrary elements are shown here. **b** The inverse phase determined from simulations is applied to each element, resulting in zero phase at the treatment location for maximum constructive interference with greater intensity than in the uncorrected case

For phase correction, HAS is used to calculate the 3D pressure patterns throughout the entire model volume from each individual element of the phased-array transducer, initially assuming zero phase and uniform amplitude, as shown in Fig. 1a. Phase offsets for aberration correction are created by taking the negative of the phase from each element as found at any given treatment location. When this negative phase is impressed on each individual element, maximum constructive interference occurs at the desired point, as indicated in Fig. 1b. Arbitrary treatment locations can be handled by saving the phases over the entire treatment volume. There is negligible additional computational cost for multiple treatment sites because the 3D pressure pattern for the treatment volume has already been computed. Currently, the algorithm only accounts for the phase differences between elements and does not alter the amplitude of the elements when steering or at the geometric focus. However, since amplitudes can be saved, it is possible to implement more advanced methods such as those presented in [25] or [21].

For fast computation that would be required in a clinical setting, each element’s pressure pattern is computed in parallel on a NVIDIA Tesla GPU (NVIDIA, Santa Clara, CA) using Jacket (ArrayFire, Atlanta, GA) and MATLAB 2012 (MathWorks, Natick, MA). For small acoustic models (229 × 159 × 182 voxels with six tissue types and 256 transducer elements), pressure patterns and phase corrections can be computed in approximately 45 s. Models such as the more clinically relevant skull flap model (421 × 648 × 170 voxels with 3000 distinct types of bone and 256 elements) take approximately 15 min for pressure patterns and corrections.

### Aberrator model

**Fig. 2:**
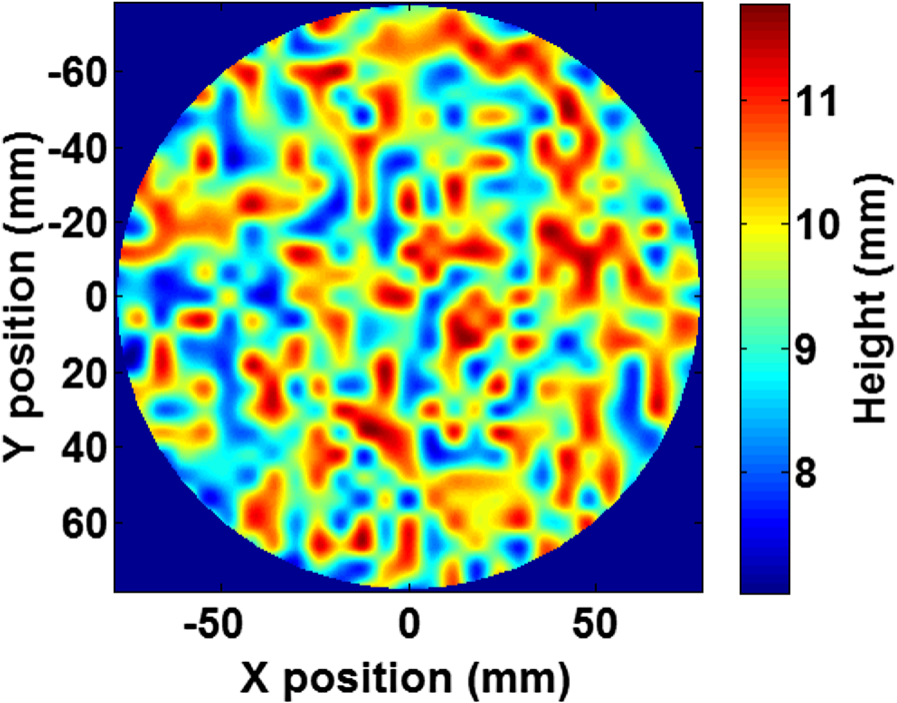
Phase aberrator height map variations in height of 3D-printed aberrator model. Heights were randomly generated to create phase shifts varying from 0 to 2π based on the speed of sound of the photopolymer used in printing (see Table 1)

Evaluation of the phase correction method can be difficult in an actual skull where there are uncertainties associated with determining the acoustic properties. To evaluate the effectiveness of the phase correction method independently of these errors, an aberrator model was developed that was made of 3D-printed photopolymer (VeroWhitePlus, Stratasys, Eden Prairie, MN). It was flat on one side and had randomly varying heights on the other side, as shown in Fig. 2. The heights on the aberrating side varied a maximum of 4 mm on top of a 7.5-mm base (giving a range of 7.5–11.5 mm total thickness). The variance in height was chosen to create phase shifts up to 2π, given the speed of sound of the plastic (Table 1). A separate rectangularly shaped block was used to determine the acoustic properties of the plastic by a through-transmission measurement [26].

**Table 1:** Acoustic properties of phase aberrator model

Speed of sound	Attenuation
2492 m/s	4.72 dB/(cm MHz)

Acoustic properties of the 3D-printed aberrator model as measured by a through-transmission test

### Ex vivo skull model

To assess what level of corrections could be expected in an actual skull, an ex vivo section of human skull approximately 15 cm × 10 cm in size was evaluated. The skull flap was discarded, de-identified, and obtained from a deceased patient, and therefore IRB exempt. It was cleaned and frozen several months before being imaged in a clinical CT scanner at a resolution of 0.46 × 0.46 × 0.3 mm. An acoustic model of the skull was built using the CT images and a published method of mapping CT Hounsfield units (HU) to acoustic properties (speed of sound, density, and attenuation). The method, described in Pichardo et al. [27], maps a linear relationship between density and HU. The speed of sound and attenuation are determined from HU using a series of curves that were created by optimizing simulations to match experimental data. Before phase correction experiments, the skull flap was rehydrated overnight inside a degasser to remove air.

### Hydrophone scans

**Fig. 3:**
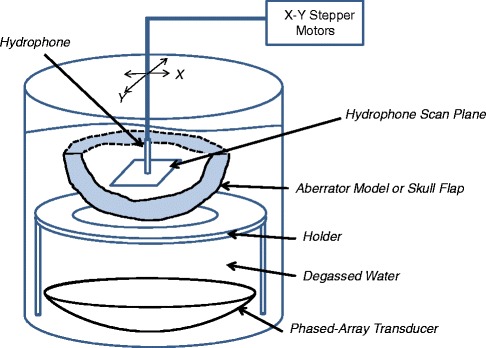
Experimental setup. Schematic of the experimental setup of hydrophone scanning system. The transverse plane was orthogonal to the direction of acoustic propagation (i.e., the transverse plane was horizontal)

Experimental verification of the phase correction method was performed using a 1-MHz 256-element phased-array transducer (IMASONIC SAS, Besançon, France) driven by high-power generators (IGT, Bordeaux, France). The transducer had a circular aperture of 14.5 cm in diameter and a focal length of 13 cm. A custom holder was built to hold the aberrator model and the skull flap at a fixed, known distance and rotation angle relative to the transducer. The distance and rotation of the holder were determined by placing the transducer and holder in an MR imager and performing a 3D GRE scan with 1-mm isotropic resolution. The resulting image was zero fill interpolated down to 0.25 mm isotropic voxel spacing, resulting in a misregistration error of 0.25 mm or less in each direction. Pressures were measured by scanning a hydrophone (HNR-0500, Onda Corporation, Sunnyvale, CA) in a raster pattern in the plane perpendicular to the direction of the beam propagation using two stepper motors (NRT150, Thorlabs Inc., Newton, NJ), as shown in Fig. 3. The signal from the hydrophone was passed through a 10-dB preamplifier before being recorded by a digital oscilloscope (PicoScope 4224, Pico Technology, Tyler, TX). These hydrophone scans had a resolution of 0.25 mm and covered an area of 10 × 10 mm. For phase correction, the hydrophone was left at the intended focus while each element was fired individually, allowing the phase to be recorded at that location. Later, the inverse of the recorded phases was applied to achieve the hydrophone time-reversal scans. The scans were performed both with and without phase corrections at the geometric focus.

## Results

### Aberrator model

**Fig. 4:**
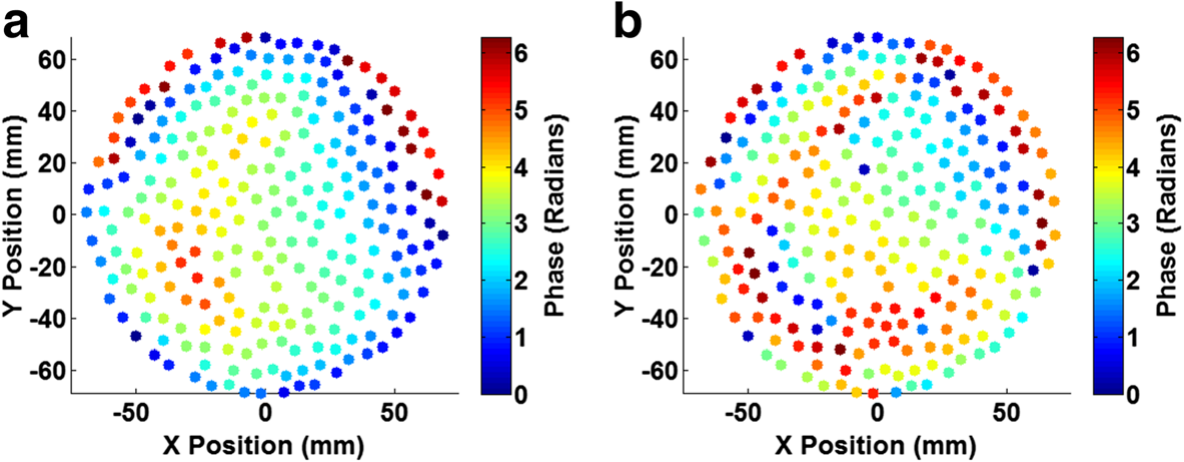
Phase maps. Computed phases obtained **a** through simulations and **b** with hydrophone measurements, for correcting aberration through the aberrator model to the geometric focus as a function of transducer element location

Simulations were run using the phase aberration correction method described in the previous section. Figure 4a shows the phases generated by the simulation-based phase correction method as a function of element location on the transducer. For comparison, the phases obtained from the hydrophone scans are shown in Fig. 4b. The time required to compute the simulation-based corrections was approximately 45 min due to the high resolution and size of the model (0.23 × 0.23 × 0.25 mm with 667 × 667 × 104 voxels), which was needed to emulate a smoothly varying aberrator.

**Fig. 5:**
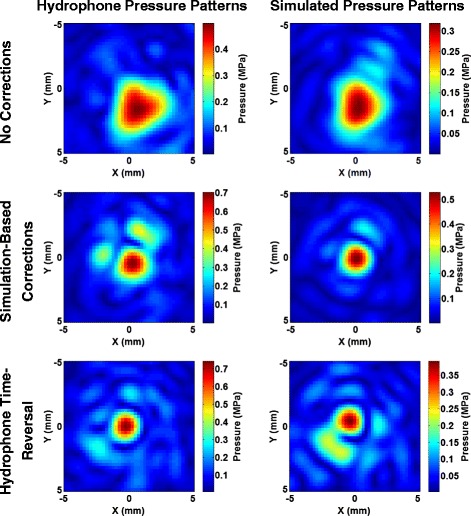
Model pressure patterns. The *left column* displays the pressure patterns at the geometric focus through the aberrator model obtained from the hydrophone scans. The *right column* displays the results of the simulated pressure patterns using the acoustic parameters listed in Table 1. The *rows from top to bottom* represent the cases of uncorrected, corrected using simulated phases calculated with known acoustic parameters, and corrected using phases obtained from hydrophone measurements, respectively

Pressure patterns at the geometric focus were obtained both with numerical simulations and with experimental hydrophone scans. As expected, the aberrator model created significant distortions in the pressure patterns in the absence of phase correction. Both the simulated and experimental pressure patterns demonstrated that without phase correction, the beam pressure was spread over a large area and displaced from the intended target, as shown in Fig. 5, upper row.

Figure 5, middle and bottom rows, shows the corrected experimental and simulated pressure patterns though the aberrator model using phases found from both the hydrophone measurements and the simulation-based method.

Table 2 gives the relative increase in maximum pressure from the hydrophone scans due to the phase correction and the distance from the point of maximum pressure to the intended treatment location. Quantitatively, the observed peak beam pressure was increased by a factor of 1.41 when simulation-based corrections were applied. Phase corrections obtained with the hydrophone measurements resulted in a 1.50-fold peak pressure increase. Moreover, the distance of the maximum pressure from the intended treatment location was reduced from 1.9 to 0.56 mm or less in both cases.

**Table 2:** Phase correction improvements in the aberrator model

	Ratio of maximum pressure corrected to uncorrected	Distance from focus (mm)
Uncorrected	1	1.90
Corrected, simulation-based	1.41	0.56
Corrected hydrophone-based	1.50	0.25

Ratio of the highest pressure with phase corrections compared to the uncorrected case and target offset distance for the aberrator model as measured by hydrophone scans

### Skull flap

**Fig. 6:**
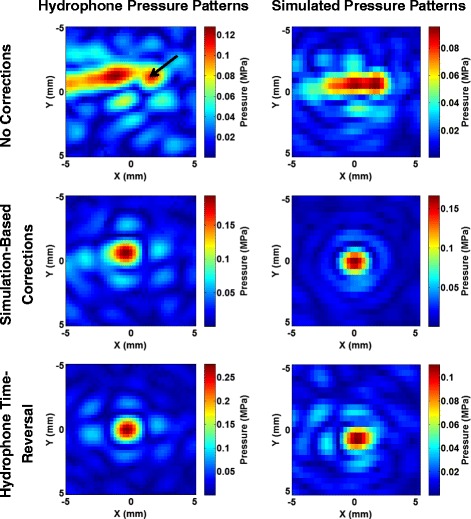
Skull flap pressure patterns. The *left column* displays the pressure patterns obtained with the hydrophone at the geometric focus through the skull flap. The *right column* displays the simulated pressure patterns*.* The *rows*, *from top to bottom*, represent the patterns with no corrections, corrections using phases obtained from simulations, and corrections using phases obtained from hydrophone measurements, respectively. The *arrow* in the uncorrected hydrophone scan points to a side lobe with 84 % of the peak main lobe pressure

Without phase correction, the ex vivo skull flap produced significant aberrations when the intended focus was at the geometric focus, as shown in Fig. 6, upper row. Displacement of the beam from the intended treatment location and spreading of the beam pattern were observed. Additionally, significant secondary side lobes were observed. An arrow in Fig. 6 indicates a side lobe with 84 % of the peak pressure of the main lobe for the hydrophone scan with no corrections.

After applying simulation-based phase corrections, significant improvements in the beam focus were observed. The computation time to compute the phases required for correction using simulations was approximately 15 min.

Quantitative data depicting the pressure levels and distance from the intended target from the hydrophone scans are listed in Table 3. The maximum pressure was increased by a factor of 1.51 for targeting at the geometric focus using simulation-based corrections. Additionally, the distance of the maximum pressure from the intended treatment location was reduced, the beam profile was smaller, and the secondary lobes were diminished. Hydrophone corrections resulted in a 2.17-fold increase in peak pressure compared to the uncorrected case.

**Table 3:** Phase correction improvements in ex vivo skull

	Ratio of maximum pressure corrected to uncorrected	Distance from focus (mm)
Uncorrected	1	1.77
Corrected, simulation-based	1.51	0.71
Corrected, hydrophone-based	2.17	0.25

Ratio of the highest pressure with phase corrections compared to the highest uncorrected pressure and target offset distance for the skull model as measured by hydrophone scans

**Fig. 7:**
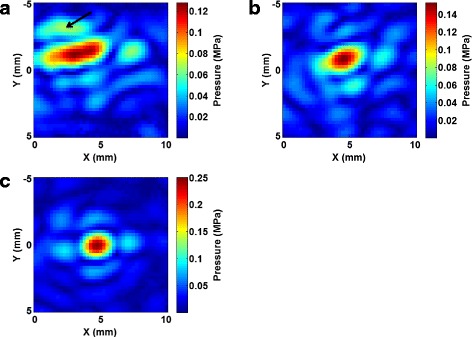
Steering pressure patterns. Hydrophone scans of the phase correction method applied to the ex vivo skull flap with beam steering at a location 5 mm transverse to the geometric focus. The intended treatment location is at 0 mm in *y* and 5 mm in *x*. **a** Hydrophone scans with no phase correction. **b** Corrections using simulated phases. **c** Correction using hydrophone-measured phases. The *arrow* indicates a primary side lobe with 54 % of the main lobe pressure

The phase correction method can also be used to target multiple treatment locations with a negligible increase in computational costs. Figure 7 shows the phase correction applied to a location 5 mm transverse to the direction of the ultrasound propagation. Uncorrected, the pressure pattern showed a side lobe with 54 % of the peak pressure of the main lobe, as indicated by an arrow in the figure. Additionally, the distance of the maximum pressure from the intended treatment location was 2.34 mm. After correcting using phases from simulations, a peak pressure 119 % larger than the uncorrected case was found at a distance of 0.9 mm from the intended focus.

### Further simulation-based analysis

The HAS method combined with phase correction allows for rapid evaluation of a number of different parameters. Of particular clinical interest are the patient-specific responses to treatments—including the ability to electronically steer the beam while maintaining adequate heating capability and the sensitivity of the system to errors in misregistration.

**Fig. 8:**
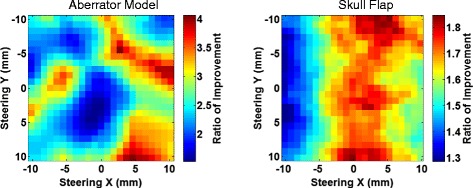
Ratio of steering improvement. Point-wise ratio of improvement of maximum pressures in the focal plane for the aberrator model (*left*) and the skull flap (*right*). Each point represents the ratio of the maximum corrected to uncorrected pressures in the focal plane when steering to a particular *x*, *y* location

Figure 8 shows the point-wise improvement when the beam is electronically steered for both the aberrator model and the skull flap. For each point, a simulation was performed using uncorrected and corrected phases when steering. This figure compares the ratio of the maximum pressure in the focal plane of the corrected to the uncorrected simulations as a function of the extent of steering.

**Fig. 9:**
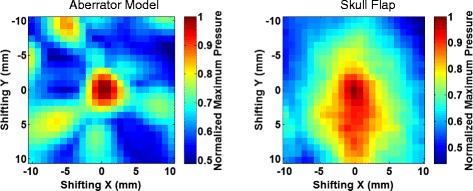
Simulated misregistration sensitivity. Simulated sensitivity of the aberrator model (*left*) and skull flap (*right*) to translational misregistration. Each pixel represents the maximum pressure in the focal plane when the model has been shifted in a plane perpendicular to the direction of beam propagation by the specified distance

Figure 9 shows the sensitivity of both the aberrator model and skull flap to misregistration using simulations with phase correction to the geometric focus. For each point, the model was translated by a specified amount in the plane perpendicular to the direction of the beam propagation while the phases were kept for the focus to mimic the effects of a misregistration. The maximum pressure in the focal plane for the shifted case was then compared to the maximum pressure assuming no shifting (i.e., at shift position 0, 0). In Figs. 8 and 9, the ratios were chosen to demonstrate the improvement independent of the power of transducer.

Although the results here cannot be generalized to other skulls, they provide a proof-of-concept of the ability of HAS in combination with phase correction procedures to rapidly simulate possible treatment scenarios. Each graph in Figs. 8 and 9 represents 529 individual simulations and can be created in less than 4 h using a Windows computer with an i5-core processor and 16 GB of RAM using MATLAB 2015a. Note that these simulations were not run in parallel—doing so would provide additional speed. Each individual simulation took approximately 20 s for the skull flap and 25 s for the aberrator model.

**Fig. 10:**
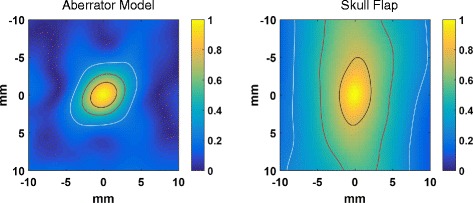
Autocorrelation of path lengths. The normalized autocorrelation of the phase lengths for the aberrator model (*left*) and skull flap (*right*) along the axis of the ultrasound propagation. *Contour lines* are displayed for values of 25 % (*white*), 50 % (*red*), and 75 % (*black*) of the maximum correlation

As a demonstration of the spatial variation in the propagation phases is related to the amount of misregistration, Fig. 10 shows the autocorrelation of 2D phase-length patterns for both the aberrator model and the skull flap. For this figure, the phase-length patterns were calculated by accumulating the phase shift encountered along the paths parallel to the axis of the ultrasound propagation. (For the skull flap, the phases were smoothed using a Gaussian kernel with a 2-pixel standard deviation to account for the CT measurement noise.) The phase lengths were then converted to phasor notation to best represent the potential for interference. A circular section of each pattern equal in size to the beam profile when it encountered the model was correlated with the untruncated pattern and normalized. This is representative of the overall spatial correlation between the phases required for phase correction.

## Discussion

The phase correction method introduced here was able to rapidly calculate the phases required to correct for aberrations. In the case where the acoustic properties were fully known (for the 3D-printed phase aberrator model), the simulation-based correction was able to recover 95 % of the peak pressure achieved with hydrophone time reversal for targeting the geometric focus (as seen in Table 2). The relatively small difference between the simulation-based and hydrophone-based corrections for the aberrator model (95 % of the peak pressure was recovered) is most likely due primarily to registration errors. The simulations require close matching between the simulated and experimental placement of the aberrator with respect to the transducer elements. Misregistration will lead to poorer corrections. Figure 9 demonstrates this effect with translational errors in a single plane. Relatively small misregistrations can cause a significant decrease in the pressures seen. For example, simulations on the misregistration for the aberrator model show that a 1-mm translation of the model results in a 1.1–7.8 % loss in the peak pressure. Misregistration causes less of an effect for the skull model, which has smoother variations over the area through which the beam travels. Figure 10 further demonstrates this fact. When there is longer correlation distance for the phase lengths, one would expect misregistration in that direction to be less disruptive, a factor for phase correction. The correlation pattern for the skull flap shows longer correlation distances along the *y*-axis, which agrees with the data in Fig. 9 showing more tolerance to misregistration along that axis. Furthermore, the skull flap displays broader areas of correlation than the aberrator model, again agreeing with the results in Fig. 9 that show that the skull flap is more tolerant to misregistration overall. The 50 % contour in the results for the skull flap encompasses an area of 205 mm^2^ while the area for the aberrator model covers 24 mm^2^.

The hydrophone time-reversal method is performed experimentally with the aberrator in place and is not subject to the registration requirements. However, there may be slight errors in the location of the transducer element positions or the power output of each element. We estimate a misregistration error of less than 0.25 mm in each direction for these experiments. Data from the aberrator model, where the acoustic properties are homogeneous and known, agree with this: 95 % of the pressure is recovered, which also agrees with the simulated misregistration data presented in Fig. 9.

The 3D-printed aberrator model introduced here is a useful tool for assessing the ability of phase correction methods in the absence of acoustic parameter uncertainties. In the skull flap, where acoustic parameter uncertainties are present, simulation-based corrections were only able to recover 70 % of the pressure achieved using hydrophone time-reversal when targeting to the geometric focus (and 61 % when steering) compared to 95 % in the aberrator model. The aberrator model could be used to evaluate other possible methods of phase correction (ray tracing or FDTD time-reversal, for example) and to compare using different transducers or setups without needing to account for possible errors in acoustic modeling.

The difference between the percentage of pressure recovered using the simulation-based and hydrophone time-reversal methods of phase correction in the aberrator model (95 %) and the human skull flap (70 %) demonstrates the necessity for accurate acoustic parameter estimation of the skull. In transcranial HIFU, a large barrier to treatments is the necessary power required to achieve sufficient heating at the focal location without causing damage to unintended locations. Although the acoustic model of the skull flap used in this paper showed a 1.52-fold increase in peak power over uncorrected results, the hydrophone time-reversal phase correction demonstrated a 2.17-fold improvement. This suggests that there may be benefit in further research into acoustic parameter estimation of the skull. The aberrator model parameters were determined via a through-transmission test on a rectangular block of the same material used to 3D print the model, while the bone was modeled using the method presented in [27], which assigned values based on CT images. However, there is some evidence that the method presented in [27] may not be accurate in all cases as simulations using the method deviated in temperature by 24 % compared to the observed data, and the peak focal point distance between the simulations and experimental measurements was off by an average of 1.6 mm [28].

Other non-invasive methods have shown recovery of 14–58 % of peak intensity of hydrophone-based corrections using human skull flaps [29] compared to this method’s recovery of 49 % of the peak intensity (70 % of the peak pressure) at the geometric focus. However, caution must be used in directly comparing results for different transducer setups and skulls. Larger aperture size will usually cause the ultrasound to travel through a larger, more inhomogeneous region, yielding a greater need for phase correction. Additionally, a change in frequency of the transducer will change the precision required for phase correction. Unquantified variations in acoustic properties between human skulls also make direct comparisons difficult. Ideally, phase correction methods should be evaluated on the same system using an aberrator model similar to the one used here to evaluate effectiveness absent these disparities.

The method introduced here is more computationally efficient when compared to other methods presented in the literature. Simulations on a GPU for the skull flap modeled with clinically relevant resolution were performed in approximately 15 min. This computational time is for the full 3D volume for each individual element, allowing for corrections at multiple treatment locations. This computational time represents an eightfold improvement over a similar FDTD-based time-reversal simulation that was performed for only a single treatment location [19].

The method described has the benefit of simulating a full 3D volume. While we have used the phase information to demonstrate the possibility for multiple treatment locations, it would also be possible to use the amplitude information for predicting off-focus hot spots. For transcranial treatments, this could allow risk assessment of skin burns or heating in important brain structures. Combining the phase correction method with rapid simulations could allow for many clinically valuable insights, including determining which patients may not be suitable for transcranial HIFU treatments, treatment envelope evaluation, establishing limits for the maximum allowable power, or treatment planning.

## Conclusions

We have presented a simulation-based method for phase correction that allows for improved focusing in transcranial HIFU applications. The method is rapid; phases can be computed in approximately 15 min for the resolution required for transcranial applications, and it creates corrections for a 3D volume allowing for treatment planning. The method was tested on both a 3D-printed acoustic aberrator and an ex vivo human skull flap where it resulted in increased pressure, decreased beam width, and reduction in side lobes. Corrections were achieved for both targeting to the geometric focus and steering to a target 5 mm away from the focus. Additionally, the method of corrections was combined with fast ultrasound simulations to evaluate the effects of steering and transducer/model misregistration on pressure levels. Since simulations are performed for a 3D volume, future work will include predicting maximum safe power usage and off-focus hot spots produced during treatments.

## Abbreviations

FDTD: Finite-difference time-domain
HAS: Hybrid angular spectrum
HIFU: High-intensity focused ultrasound
HU: Hounsfield units
